# Electroacupuncture Stimulation Suppresses Postoperative Inflammatory Response and Hippocampal Neuronal Injury

**DOI:** 10.1155/2022/3288262

**Published:** 2022-09-06

**Authors:** Xiang-feng Wang, Qin Lin, Guan-hui Wang, Gen-ming Zhan, Wei Liu, Zhi-wei Lin

**Affiliations:** Department of Anesthesia, People's Hospital Affiliated to Fujian University of Traditional Chinese Medicine, Fuzhou 350004, China

## Abstract

Postoperative cognitive dysfunction (POCD) is consequence of anesthesia and surgery that primarily affects older people. The prevention and treatment of POCD has drawn an increasing attention in recent decades. Here, we established the animal model mimicked POCD after femoral fracture surgery, and analyze the effect of acupuncture stimulation on postoperative cognitive function after femoral fracture surgery. Compared with the mock group, the cognitive function performance was significantly decreased both in the anaesthesia group and the surgery group, between which the symptoms were more severe in the surgery group. The peripheral inflammation response and the neuron impairment and inflammation response in the hippocampus were observed in the surgery group, but only peripheral inflammation response was detected in the anaesthesia group. These findings indicated the POCD was the synergistic outcome of anaesthesia and surgical stimulation but with different pathogenic mechanism. The surgery with mental tri-needles (surgery+MTN) group outperformed the surgery group in terms of cognitive function performance. The peripheral inflammation response and the neuron impairment and inflammation response in the hippocampus was significantly reduced by the electroacupuncture stimulation. Our findings indicated the protection of electroacupuncture form POCD after femoral fracture surgery is related to the inhibition of inflammation response and neuron impairment.

## 1. Introduction

POCD is a condition that primarily affects the elderly following surgery. Although the complication is first known in patients who underwent cardiac surgery, increasing evidences demonstrate the POCD also widely occurred after noncardiac surgeries [1]. The symptoms of POCD are varied and include dysfunction in consciousness, attention, memory, orientation, and executive function. POCD affects patients' daily life, work, and activities. In a serious condition, POCD may persist for years postoperative or, worse, become a permanent disorder [2, 3]. Patients with chronic POCD, in consequence, require additional assistance in daily life and probably have to leave office or retire, leading to a great decrease in the quality of life; besides, long-term POCD is more likely associated with the increased mortality after surgery [3–5]. Given the great number of surgeries in the elderly [6], POCD prevention and treatment have become a global concern.

The pathological and pathogenic mechanism of POCD is still intricate and inconclusive, despite strenuous efforts in decades. According to previous findings, POCD is related to neuroinflammation, oxidative stress, mitochondrial dysfunction, blood brain barrier disruption, hippocampal synaptic damage, and autophagy disorder [7–11]. Increasing evidences indicate neuropsychiatric disorder may be the root cause of POCD. The neuropsychiatric disease, Alzheimer's disease, likely increases the risk of POCD, and amyloid *β* deposition and tau protein phosphorylation, pathological characteristics of Alzheimer's disease, are observed in POCD patients [12, 13]. Preoperative stress and/or depression appears to increase the risk of POCD, and vice versa [14–16]. Lin et al. [11] suppose that the peripheral immune reaction can cause neuroinflammatory response, following the damage of cerebral tissue, and the impairment of microglia, astrocytes, and mast cells likely result in the development of POCD. These findings imply that surgery probably causes cerebral tissue impairments and the development of POCD. Therefore, the reduction in damage of cerebral tissue may probably be the principal strategy to prevent the development of POCD.

Acupuncture is a sophisticated medical therapy that has been used for a thousand years in China. Acupuncture is widely applied to treatments in brain injuries, psychological illnesses, pain syndrome illnesses, and gastrointestinal disorders, because of its broad-spectrum bioactivity in nervous system, immune system, metabolism, and gastrointestinal system [17]. In recent decades, acupuncture has been increasingly used in surgical patients for POCD prevention and treatment. Acupuncture stimulation substantially alleviates the illness after surgery and effectively accelerates recovery rate in POCD patients [18, 19]. For the acupuncture stimulation in pre- and/or perioperation, the incidence of POCD appears to be decreased and the POCD impairment is lightened compared with the control group, and a higher recovery rate is observed in acupuncture-treated patients [20–22]. Some studies, in contrast, demonstrate that acupuncture stimulation does contribute to alleviate the POCD impairment and increase recovery rate in pre- and/or perioperation, but no effect is observed on the incidence of POCD [23]. The discrepancy in acupuncture effect may result from different types of surgeries.

The information on effect mechanism of acupuncture therapy on POCD is predominantly derived from the rat model of POCD after hepatectomy. As mentioned above, the effect and mechanism of acupuncture on POCD patients may be surgery-specific [24]. Here, we established the animal model of POCD after femoral fracture surgery, which is frequently performed in the elderly. The whole goal in the present study was to understand effect and mechanism of acupuncture on POCD in the elderly after femoral fracture surgery. The specific objectives were (1) to describe the effect of acupuncture stimulation on postoperative cognitive performance, (2) to illustrate the effect of acupuncture stimulation on histological morphology and expression level of inflammation-related genes in hippocampus, and (3) to elucidate the effect of acupuncture stimulation on serum concentration of inflammation-related genes.

## 2. Materials and Methods

### 2.1. Animals

Shanghai Sippr BK laboratory animal Co. Ltd. provided sixty adult male Sprague-Dawley rats (clean-grade, 20 months old, *ca.* 500-600 g in weight) which were randomly assigned to one of four groups: mock, anaesthesia, surgery, and surgery+MTN. The anaesthesia group received anaesthesia injection without surgery, the surgery group received femoral fracture surgery, and the surgery+MTN group received electroacupuncture stimulation pre- and perioperative using MTN. Rats were separately housed in a cage exposed to natural light, of which the ambient temperature was at approximately 23°C, and were acclimated for a week prior to experiments.

#### 2.1.1. Surgery Protocol

The femoral fracture surgery were performed according to the description of Holstein et al. [25]. In brief, the rats were sedated with 0.25 ml/100 g of 2% sodium pentobarbital administered intraperitoneally. The skin and muscle at the proximal end of the right femur were incised layer by layer to expose the femur and drilled along the direction of the femoral neck, with a depth close to the articular cartilage. After drilling, the needle (diameter = 0.4 mm) was removed; the femur was subsequently fractured by the bending device, followed by the insertion of a screw (1.7 × 12 mm). The wound was sutured layer by layer and disinfected with iodophor.

**Figure 1:**
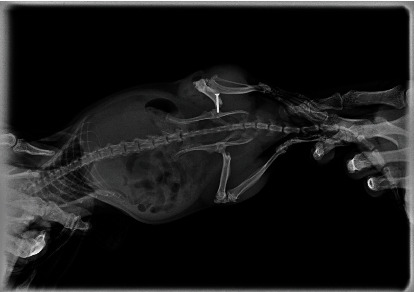
The rat model of POCD after femoral fracture surgery. The femur was fractured, and a screw was inserted into the canal.

The entire operation time was about 30 min. After the operation, the incision was infiltrated with 0.25% bupivacaine, sutured, and fixed. After the animals were awake, they were put back into a cage covered with cotton gauze pads and kept warm. After the operation, 1% vital iodine was applied to the wounds of rats twice a day until the wounds healed. During the experiment, no rats died, and screws were clearly seen through X-ray detection, indicating that the modeling was successful (Figure 1).

### 2.2. Electroacupuncture Stimulation

In the surgery+MTN group, the electroacupuncture stimulation was performed on rats following anaesthesia prior to the surgery, according to the description of Liu et al. [26]. Briefly, stainless acupuncture needles (0.26 mm × 15 mm) were vertically inserted (*ca*. 2.5 mm in depth) into three acupoints, including Shenting (DU24) located in the anterior midline of the rat's head, in front of the junction of the fronto-parietal suture, and two Benshen (GB13) located in the middle and outer 1/3 of the line connecting the inner and outer canthus of the eye of the rat, about 3 mm straight up. Acupuncture was used in conjunction with the HANS-200A Acupoint Neurostimulator, which was programmed with a 2/15 Hz sparse-dense wave and an intensity of 0.2 mA over 20 minutes.

### 2.3. Cognitive Function Analyses

To evaluate cognitive performance, the open field and Morris water-maze tests were conducted on rats. The rat was placed in the center of a closed space for the open field test, of which floor were divided into 25 equal squares (20 cm × 20 cm), which was regularly cleaned after each trial. The duration in the central square and the number of squares crossed in 3 min were recorded.

The rat was placed in a plastic circular swimming pool, which was divided into four quadrants based on 4 points on the north, south, east, and west of the rim, and a depth of 25 cm with water was filled in the pool for the Morris water-maze test (Supplementary 1 video training, https://drive.google.com/file/d/1sybDOGYECVn8nLsbbOm-cm2qUxpoZx75/view?usp=sharing). In the center of pool's quadrant I, a transparent platform (diameter = 10 cm) was submerged under a 1 cm layer of water. The rat was introduced into the pool randomly from the 4 points. Escape latency was calculated as the time it took to get from the introduction point to the platform. The escape latency was recorded as 60 s, if the rat did not climb onto the platform within 60 s. The escape latencies from the 4 immersion points were averaged for each rat. The platform was removed once the latency trial was completed, and the animal was put in quadrant III of the water maze. Over the course of 60 seconds, the swimming path and time spent in quadrant I, as well as number of platform crossings, were recorded. All of the trials were also filmed for later examination.

### 2.4. Sampling

After the completion of cognitive function tests, blood was drawn from the tail vein and centrifuged for 10 min at 2,600 g to obtain plasma. After blood collection, the rat was given a lethal dosage of pentobarbital and the brain was dissected immediately. The hippocampus was sampled and was stored in -80°C freezer until following analyses, as well as plasma sample. In addition, the brain sample was fixed with 4% paraformaldehyde for 24-48 h.

#### 2.4.1. Histological Analyses

The fixed brain sample was subsequently undergone gradient dehydration, embedded into paraffin, and was continuously sectioned (5 *μ*m thickness), followed by deparaffinization with xylene and HE staining to make slides. The slides were observed under the light microscopy. To determine apoptosis frequency, the slides were TUNEL stained with in situ cell death detection POD Kits (Roche, Penzberg, Germany), as directed by the manufacturer. The TUNEL-positive cells with clear nuclear labeling were counted and the percentage of TUNEL-positive cells was calculated: (the number of apoptotic cells/total number of nucleated cells) × 100.

#### 2.4.2. ELISA, Western Blot, and qPCR

The plasma concentrations of IL-1, IL-6, and TNF-*α* were determined with corresponding ELISA kits according to the manufacturer's instructions. The results were measured by a Microplate Reader (Thermo Scientific Microplate Reader) at an absorbance of 450 nm.

Proteins were extracted from hippocampus samples using RIPA lysis solution containing a cocktail of protease and phosphatase inhibitors as well as PMSF (Beyotime, Shanghai, China). 10% SDS-PAGE gel was used to separate protein which was then transferred to PVDF membranes (Bio-Rad, Hercules, CA, USA). After blocking with 5% nonfat milk in TBST (Tris-buffered saline containing 0.1% Tween 20), the membrane was incubated overnight at 4°C with a monoclonal rabbit anti-IL-1*β*, anti-IL-6, and anti-TNF-*α* primary antibodies, and GAPDH (Cell Signaling Technology) was used as an internal control, followed by washing in TBST. The washed membrane was incubated for 1 hr at room temperature with respective HRP-conjugated secondary antibodies. The Immun-StarTM HRP Chemiluminescence Kit (Bio-Rad) and the ImageQuant LAS 4000 system (GE Healthcare, Hino, Japan) were used to view the protein bands and capture the images, respectively. ImageJ analysis software was used to calculate the intensity of protein bands.

LightCycler 480 equipment (Roche, Switzerland) and SYBR Green PCR MasterMix to measure the levels of *IL-1β*, *IL-6*, and *TNF-α*. For the PCR templates, first-strand cDNA was reverse-transcribed by DNase I treated total RNA which was isolated from the hippocampus samples using Trizol reagents (TaKaRa). The PCR program was set as following: a preheating at 95°C for 60 s, 40 repeated cycles of heating at 95°C for 30 s, cooling at 58°C for 35 s, and extension at 72°C for 60 s and a final extension at 72°C for 10 min. The relative expression level was estimated using the 2^−*ΔΔ*CT^ algorithm. The PCR primers are listed in Table 1.

### 2.5. Statistical Analysis

In software SPSS 19, for group comparisons, a one-way ANOVA was employed, with Tukey's post hoc test for pair comparisons. The Shapiro-Wilk test was used to ensure that the data was normal before undertaking parametric tests. When *P* < 0.05, a significant difference was regarded.

## 3. Results

### 3.1. Cognitive Function Performance

**Figure 2:**
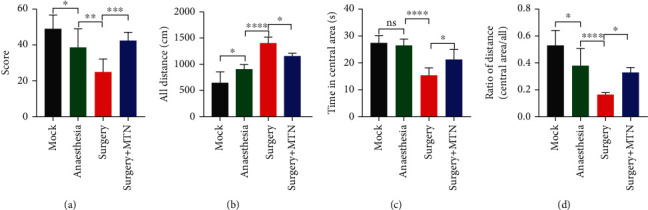
Comparisons of cognitive function performance of rats among different groups. The rats were divided into the mock group, anaesthesia group, surgery group, and surgery+MTN group. The cognitive function performance was evaluated using open field test ((a) score) and Morris water-maze test ((b) all distance, (c) time in central area, and (d) ration of distance). To compare the cognitive function performance among groups, one-way ANOVA was used in combination with Tukey's post hoc test. ^∗^*P* < 0.05,  ^∗∗^*P* < 0.01,  ^∗∗∗^*P* < 0.001, and^∗∗∗∗^*P* < 0.0001.

In the open field test, the mock group's score (mean ± SD = 48.88 ± 7.61) was considerably higher than the anaesthesia group's (mean ± SD = 38.50 ± 10.30), which was significantly higher than the surgery group's (mean ± SD = 24.88 ± 7.14) (*F* = 14.70, *P* < 0.05, Figure 2(a)). In the water-maze test, significant difference was observed in all comparisons of all distance between groups (*F* = 37.15, *P* < 0.05, Figure 2(b)), and the mean of all distance was 1410.00 cm (SD 106.4 cm) in the surgery group, 1160.00 cm (SD 51.57 cm) in the surgery+MTN group, 911.00 cm (SD 88.05 cm) in the anaesthesia group, and 655.9 cm (SD 206.90 cm) in the mock group. There is no difference observed in the time in central area between the mock group (mean ± SD = 27.29 ± 2.81) and the anaesthesia group (mean ± SD = 26.50 ± 2.17), which both were significantly longer than that in the surgery+MTN group (mean ± SD = 21.14 ± 3.67) that was significantly longer than the time in the central area of the surgery group (mean ± SD = 15.40 ± 2.61) (*F* = 20.14, *P* < 0.05, Figure 2(c)). The ratio of distance of the surgery group (mean ± SD = 0.1676 ± 0.01) was observably lower than that of other groups, and the ratio of distance of the mock group (mean ± SD = 0.5314 ± 0.11) was markedly increased compare to the anaesthesia group (mean ± SD = 0.3820 ± 0.13) and the surgery+MTN group (mean ± SD = 0.3303 ± 0.04), between which there was no difference (*F* = 17.52, *P* < 0.05, Figure 2(d)).

### 3.2. Histological Observation

**Figure 3:**
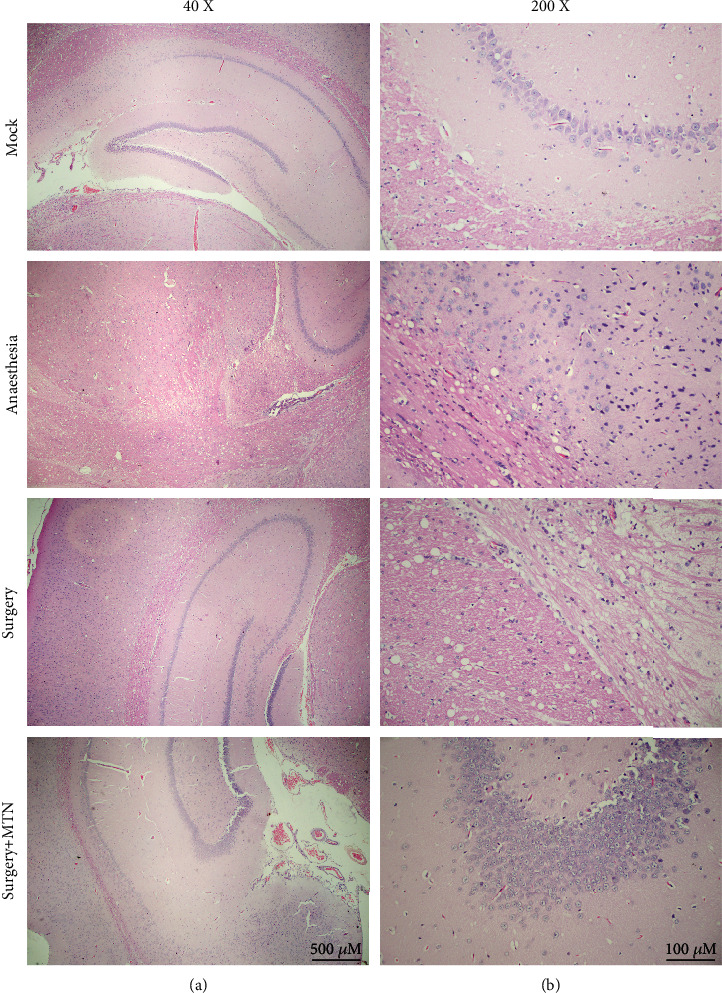
Comparisons of histological morphology in the hippocampus among different groups. The rats were divided into the mock group, anaesthesia group, surgery group, and surgery+MTN group. The HE staining sections were observed under the light microscopy ((a) 40x, (b) 200x).

Intact cells in regular shape were observed in hippocampal neurons of the mock group (Figure 3). Compared with the mock group, hippocampal neurons were most seriously damaged in the surgery group, followed by the anaesthesia group. Compared with the surgery group, the morphology of hippocampal neurons appeared to be much normal in the surgery+MTN group. The surgery group (mean ± SD = 47.87% ± 11.07%) had a considerably higher percentage of TUNEL positive cells than the surgery+MTN group (mean ± SD = 21.63% ± 6.81%).

**Figure 4:**
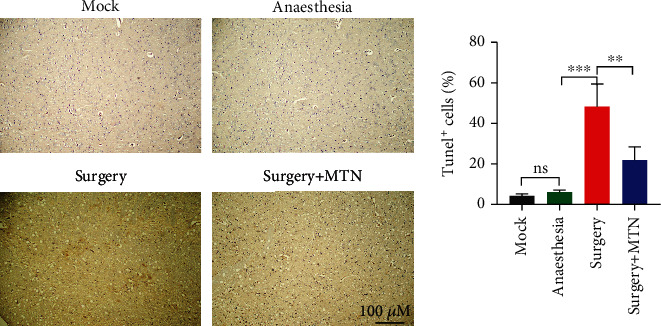
The comparison of TUNEL-positive cells in the hippocampus among different groups. The rats were divided into the mock group, anaesthesia group, surgery group, and surgery+MTN group. The percentage of TUNEL-positive cells was calculated. To compare the percentage of TUNEL-positive cells among groups, one-way ANOVA was used in combination with Tukey's *post hoc* test. ^∗^ *P* < 0.05,  ^∗∗^*P* < 0.01,  ^∗∗∗^*P* < 0.001, and^∗∗∗∗^*P* < 0.0001.

The percentage of TUNEL-positive cells of the surgery group (mean ± SD = 47.87% ± 11.07%) was significantly higher than that of the surgery+MTN group (mean ± SD = 21.63% ± 6.81%), which was significantly higher than the percentage of TUNEL-positive cells of the mock group (mean ± SD = 4.133% ± 0.97%) and the anaesthesia group (mean ± SD = 6.00% ± 1.04%) (*F* = 28.70, *P* < 0.05), between which there was no difference (Figure 4).

### 3.3. Gene and Protein Expression

**Figure 5:**
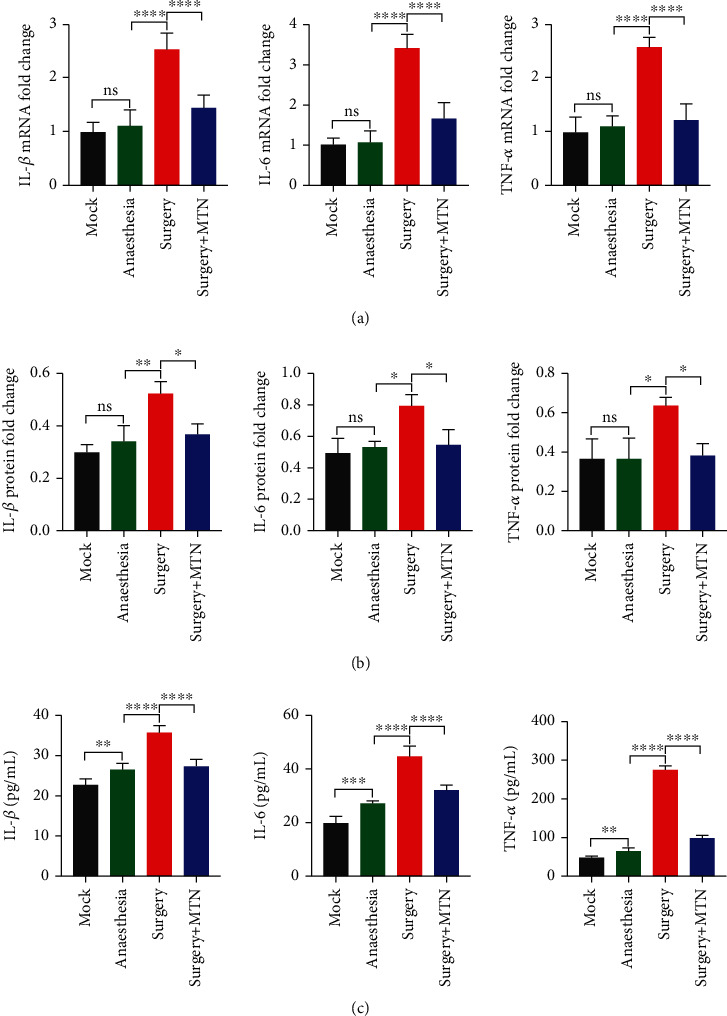
Comparisons of expression level of IL-1*β*, IL-6, and TNF-*α* genes in hippocampus and serum concentration of IL-1*β*, IL-6, and TNF-*α* among different groups. The rats were divided into mock group, anaesthesia group, surgery group and surgery+MTN group. The mRNA expression level was determined by qPCR using *β*-actin as the internal control (a). The serum concentration was measured by ELISA (b). To compare the gene expression level and serum protein concentration among groups, one-way ANOVA was used in combination with Tukey's *post hoc* test. ^∗^*P* < 0.05,  ^∗∗^*P* < 0.01,  ^∗∗∗^*P* < 0.001, and^∗∗∗∗^*P* < 0.0001.

In the hippocampus, the *IL-1β*, *IL-6*, and *TNF-α* shared a similar pattern of variations in mRNA level between groups (Figure 5(a)). The mRNA level of *IL-1β*, *IL-6*, and *TNF-α* was significantly higher than that of the surgery group (mean ± SD = 2.55 ± 0.29 for *IL-1β*, 3.42 ± 0.34 for *IL* − 6, and 2.58 ± 0.19 for *TNF-α*) than that of the surgery+MTN group (mean ± SD = 1.45 ± 0.24 for *IL-1β*, 1.67 ± 0.39 for *IL-6*, and 1.23 ± 0.30 for *TNF-α*) (*F* = 71.87, *P* < 0.05 for *IL-1β*, *F* = 125.00, *P* < 0.05 for *IL-6*, and *F* = 81.16, *P* < 0.05 for *TNF-α*). The mRNA level of *IL-1β* and *IL-6* was significantly higher in the surgery+MTN group than that of the mock group (mean ± SD = 1.00 ± 0.17 for *IL-1β* and 1.00 ± 0.18 for *IL-6*) and the anaesthesia group (mean ± SD = 1.11 ± 0.29 for *IL-1β*, 1.08 ± 0.27 for *IL-6*), between which there was no difference. In addition, no difference was observed in mRNA level of *TNF-α* between the surgery+MTN group and the mock group (mean ± SD = 1.00 ± 0.27) and between the surgery+MTN group and anaesthesia group (mean ± SD = 1.09 ± 0.21).

**Figure 6:**
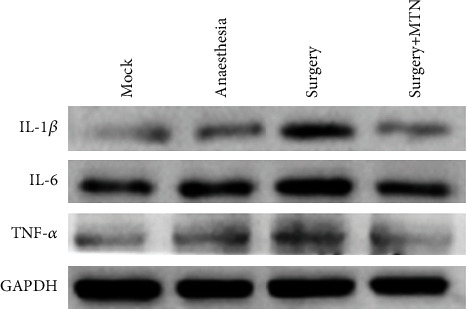
The protein levels of IL-1*β*, IL-6, and TNF-*α* in the hippocampus were detected by western blotting.

In the hippocampus, the IL-1*β*, IL-6, and TNF-*α* shared a similar pattern of variations in protein level between groups (Figure 5(b) and Figure 6). The protein level of IL-1*β*, IL-6, and TNF-*α* was significantly higher of the surgery group (mean ± SD = 0.53 ± 0.04 for IL-1*β*, 0.80 ± 0.07 for IL-6, and 0.64 ± 0.04 for TNF-*α*) than that of the surgery+MTN group (mean ± SD = 0.37 ± 0.04 for IL-1*β*, 0.55 ± 0.10 for IL-6, and 0.38 ± 0.06 for TNF-*α*), anaesthesia group (mean ± SD = 0.34 ± 0.06 for IL-1*β*, 0.53 ± 0.04 for IL-6, and 0.37 ± 0.10 for TNF-*α*), and the mock group (mean ± SD = 0.30 ± 0.03 for IL-1*β*, 0.50 ± 0.09 for IL-6, and 0.37 ± 0.10 for TNF-*α*) (*F* = 14.75, *P* < 0.05 for IL‐1*β*, *F* = 9.316, *P* < 0.05 for IL‐6, *F* = 8.032, *P* < 0.05 for TNF-*α*), between which there was no difference.

In circulation, the IL-1*β*, IL-6, and TNF-*α* shared a similar pattern of variations in concentration between groups (Figure 5(c)). The serum concentration of IL-1*β* in the surgery group was signally greater (mean ± SD = 36.16 ± 1.56) than that in the surgery+MTN group (mean ± SD = 27.75 ± 1.50), which was memorably higher than IL-1*β* concentration in the mock group (mean ± SD = 22.98 ± 1.46) (*F* = 81.90, *P* < 0.05, Figure 5(c)). The IL-1*β* concentration in the anaesthesia group (mean ± SD = 26.83 ± 1.48) was increased compared with the mock group and was not different with that of the surgery+MTN group. Significant difference was observed in all comparisons of IL-6, and TNF-*α* concentration between groups (*F* = 105.5, *P* < 0.05 for IL-6 and *F* = 1157.0, *P* < 0.05 for TNF-*α*) and the mean of serum concentration was 45.04 (SD 3.66) for IL-6 and 275.6 (SD 10.96) for TNF-*α* in the surgery group, 32.27 (SD 1.78) for IL-6 and 99.28 (SD 5.45) for TNF-*α* in the surgery+MTN group, and 27.34 (SD 0.88) for IL-6 and 65.30 (SD 8.31) for TNF-*α* in the anaesthesia group, and 19.74 (SD 2.60) for IL-6 and 49.15 (SD 2.60) for TNF-*α* in the mock group.

## 4. Discussion

The cognitive function performance was significantly decreased in the rats undergone femoral fracture surgery, compared with the mock group. The femoral fracture surgery successfully induced the POCD of rats. In these rats, the increase of serum concentration of IL-1*β*, IL-6, and TNF-*α* probably indicated the occurrence of inflammation response resulting from the femoral fracture surgery, which was in consistent with observation in the POCD elderly after femoral fracture surgery [27]. In the present study, we also found the expression levels of IL-1*β*, IL-6, and TNF-*α* genes were significantly increased in the hippocampus, which indicated the presence of neuroinflammation response. The neuroinflammation was likely associated with the neurological impairment in the POCD rats, given the increase of abnormal neurons and apoptotic cells in the hippocampus. In de facto, peripheral inflammation and related factors can trigger the inflammatory response in central nervous system [11], since peripheral proinflammatory signals can cause neurotoxic symptoms through blood-brain barrier and circulatory inflammatory factors can also regulate the systemic inflammatory [28, 29]. These findings implied the damage of central nervous system and neuroinflammation probably be related to the peripheral inflammation response in the POCD rats after femoral fracture surgery, in agreement with findings from the other POCD model of frontal lobe surgery [30]. A question, subsequently, arises that whether the incidence and severity of POCD are related to the peripheral inflammation response after surgery that merits further investigations.

The cognitive function performance greatly decreased in the anaesthesia injection group, but not as severely as in the surgery group, given that the POCD in the postoperative rats could not be exclusively attributed by the surgical stimulation. These findings indicated the POCD of postoperative rats was the synergistic outcome of anaesthesia and surgical stimulation. Although the anaesthesia contributed to the development of POCD, the pathogenic mechanism of anaesthesia was likely different from the surgical stimulation. Previous findings have proven that anaesthesia contribute to the development of POCD in the elderly and the effect of anaesthesia on patients varies due to different anaesthesia agents and methods [31, 32]. Based on cell culture models, Fodale et al. [5] suggest that anaesthesia induces apoptosis and increases amyloid *β* formation of central nervous system, leading to the development of POCD similar pathological mechanism of Alzheimer's disease, but the suggestion is still absent of supports from animal model or clinical relevance [33]. In the present study, in contrast with the surgery group, the damage of hippocampal neurons and inflammation was not observed in the anaesthesia group and there is no difference in neuron morphology and apoptosis in the hippocampus between the anaesthesia group and the mock group, except for the serum IL-1*β*, IL-6, and TNF-*α*. The increase in serum concentration of IL-1*β*, IL-6, and TNF-*α* indicated the anaesthesia injection also induced peripheral inflammation response. However, the peripheral inflammation response in the anaesthesia group did not appear to affect the hippocampus as the surgery group. A possible explanation might be that the peripheral inflammation response for anaesthesia affect other region of the central nervous system but not the hippocampus. Nonetheless, pathogenic mechanism of POCD induced by anaesthesia in the elderly merits further investigations.

Compared with the surgery group, the cognitive function performance was significantly improved in the surgery+MTN group. The discrepancy indicated electroacupuncture stimulation substantially alleviated the impairment from femoral fracture surgery. The therapeutic effect of electroacupuncture has been demonstrated in the POCD patients after surgery [20, 34]. In the present study, the peripheral inflammation response, the damage of central nervous system, and neuroinflammation were significantly reduced in the surgery+MTN group compared with the surgery group. Similar findings are observed in the other POCD model of frontal lobe surgery [26, 35]. The effect mechanism of electroacupuncture is currently complicated and mysterious on the prevention and treatment of POCD. According to previous findings, a variety of effects of electroacupuncture stimulation have been demonstrated on various systems [17]. Specifically, the electroacupuncture stimulation can change the amount of neuropeptides and neurotransmitters, as well as ionic concentrations, in the central nervous system, and besides, the electroacupuncture stimulation also plays immunomodulator and lipolytic roles in the immune system metabolism, respectively. A study suggests that the electroacupuncture protection from POCD is related to the inhibition of inflammatory factor and oxygen free radical damage and the regulation of the central cholinergic system and the synaptic function [35]. Although the mechanism of electroacupuncture is still in its infancy, our findings confirmed the protective effects of electroacupuncture stimulation on POCD. The specific protocol of electroacupuncture application remains to develop for femoral fracture surgery in clinical.

## Figures and Tables

**Table 1:** Primers used in the present study.

Gene	Forward primer (5′-3′)	Reverse primer (5′-3′)
*IL-1β*	TCCAGGATAAGGACATGAGCAC	GAACGTCACACACCAGCAGGTTA
*IL-6*	AAATTCGGTACATCCTCGAC	CCTCTTTGCTGCTTTCACAC
*TNF-α*	GTTCTATGGCCCAGACCCTCAC	GGACCACTAGTTGGTTGTCTTTG
*β-Actin*	CCCATCTATGAGGGTTACGC	TTTAATGTCACGCACGATTTC

## Data Availability

The datasets used and analyzed during the current study are available from the corresponding author on reasonable request.

## Supplementary Materials

Supplementary Materials Supplementary Materials Supplementary 1 video training: the plastic circular swimming pool for Morris water-maze test. The pool was divided into four quadrants based on 4 points on the north (■), south (▼), east (●), and west (★) of the rim, and the pool was filled to a depth of 25 cm with water.
